# RETRACTION: Comparative Analysis of Surgical and Non‐Surgical Wound Approaches in Diabetic Foot Ulcer Treatment: Meta‐Analysis and Systematic Review

**DOI:** 10.1111/iwj.70434

**Published:** 2025-03-26

## Abstract

**RETRACTION:** LeiY. , JiangP. , and TianT. , “Comparative Analysis of Surgical and Non‐Surgical Wound Approaches in Diabetic Foot Ulcer Treatment: Meta‐Analysis and Systematic Review,” International Wound Journal 21, no. 4 (2024): e14601, 10.1111/iwj.14601.38158715 PMC10961902

The above article, published online on 29 December 2023, in Wiley Online Library (http://onlinelibrary.wiley.com/), has been retracted by agreement between the journal Editor in Chief, Professor Keith Harding; and John Wiley & Sons Ltd. Following an investigation by the publisher, all parties have concluded that this article was accepted solely on the basis of a compromised peer review process. The editors have therefore decided to retract the article. The authors did not respond to our notice regarding the retraction.

**RETRACTION:** Y. Lei , P. Jiang , and T. Tian , “Comparative Analysis of Surgical and Non‐Surgical Wound Approaches in Diabetic Foot Ulcer Treatment: Meta‐Analysis and Systematic Review,” International Wound Journal 21, no. 4 (2024): e14601, 10.1111/iwj.14601.38158715 PMC10961902

The above article, published online on 29 December 2023, in Wiley Online Library (http://onlinelibrary.wiley.com/), has been retracted by agreement between the journal Editor in Chief, Professor Keith Harding; and John Wiley & Sons Ltd. Following an investigation by the publisher, all parties have concluded that this article was accepted solely on the basis of a compromised peer review process. The editors have therefore decided to retract the article. The authors did not respond to our notice regarding the retraction.

